# Supplementary material for: Environmental management accounting affects bank performance with mediators
Source: PLoS One. 2025 Oct 31;20(10):e0321174. doi: 10.1371/journal.pone.0321174 (PMC12578248; doi:10.1371/journal.pone.0321174)
Supplement: Attachment — Submitted filename: R.2.docx [file pone.0321174.s001.docx]

**Reviewer , Dr.Omar ALsinglawi**

**Paper title:**

**Environmental Management Accounting Affects Bank Performance with Mediators**

The paper investigates the influence of Environmental Management Accounting (EMA) on the performance of Vietnamese commercial banks, focusing on the mediating role of environmental costs (EC), knowledge management (KM), and green innovation (GI). Utilizing a mixed-methods approach, the study employs qualitative expert interviews and quantitative Partial Least Squares Structural Equation Modeling (PLS-SEM) to analyze data from 312 respondents. The findings suggest significant positive relationships between EMA and KM, GI, and EC, as well as between these mediators and bank performance. The paper emphasizes the importance of integrating EMA into sustainable banking practices to enhance performance and environmental responsibility.

**Strengths:**

1. **Relevance and Contribution:** The study addresses a critical and underexplored topic, particularly in the context of Vietnam, where EMA practices are relatively new.
2. **Methodological Rigor:** The use of a mixed-methods approach and robust statistical tools (PLS-SEM) ensures comprehensive and credible findings.
3. **Practical Implications:** The paper offers actionable insights into how banks can integrate EMA and sustainability practices to improve performance.

**Weaknesses and Recommendations:**

1. **Introduction and Contextualization:**
   - The introduction provides a general overview but lacks a compelling narrative to highlight the research gap.
   - **Recommendation:** Clearly state the novelty of the study and how it addresses specific gaps in EMA research within the Vietnamese banking sector.
2. **Literature Review:**
   - The literature review is extensive but occasionally redundant, especially in repeating the definitions of EMA and EC.
   - **Recommendation:** Streamline the review to focus on critical theories and studies directly relevant to the hypotheses.
3. **Research Methodology:**
   - The sample size of 312 is adequate but might benefit from more diversity in bank types or geographical representation.
   - **Recommendation:** Discuss the potential impact of sample limitations on generalizability.
4. **Data Analysis and Presentation:**
   - While the statistical analysis is thorough, the presentation of results (e.g., VIF values, discriminator validity) can be made more accessible with clearer visual aids.
   - **Recommendation:** Include more graphical representations, such as path diagrams for the PLS-SEM model.
5. **Conclusion and Practical Implications:**
   - The conclusion reiterates the findings but lacks a forward-looking perspective on future research and practical applications.
   - **Recommendation:** Provide specific strategies for banks to implement EMA practices and suggest areas for further research.
6. **Language and Formatting:**
   - There are minor grammatical errors and inconsistencies in formatting.
   - **Recommendation:** Conduct a thorough language review to ensure clarity and adherence to journal guidelines.

**Recommendation:**
Accept the paper after addressing the minor revisions suggested above. The study offers valuable insights into sustainable banking practices and makes a significant contribution to the literature on EMA.
