# Supplementary material for: Environmental management accounting affects bank performance with mediators
Source: PLoS One. 2025 Oct 31;20(10):e0321174. doi: 10.1371/journal.pone.0321174 (PMC12578248; doi:10.1371/journal.pone.0321174)
Supplement: Attachment — Submitted filename: Response to Reviewers.docx [file pone.0321174.s003.docx]

PONE-D-24-30039

Environmental Management Accounting Affects Bank Performance with Mediators

PLOS ONE

| **No.** | **Reviewer 1’s comments** | **Author’s explanations** |
| --- | --- | --- |
| 1. | Introduction and Contextualization: | The author has revised the Introduction section based on the reviewer’s comments: - **Page 2 – 4.** |
|  | o The introduction provides a general overview but lacks a compelling narrative to highlight the research gap.  o Recommendation: Clearly state the novelty of the study and how it addresses specific gaps in EMA research within the Vietnamese banking sector. | **1. Introduction**  Environmental pollution resulting from industrial activities is becoming increasingly severe. Methods for mitigating environmental pollution in business operations are of great concern to both enterprises and regulatory authorities. To address this issue, businesses need to understand the regulations, invest in upgrading waste treatment systems, and simultaneously implement environmental management accounting (EMA). Sustainable development is a process that meets current needs without compromising the interests of future generations. As a crucial and leading component of sustainable economic development, businesses are responsible for assessing and accounting for environmental factors to protect the environment.  The environment has significant importance in the realm of sustainable development. Organizations are increasingly prioritizing sustainable accounting, aiming to integrate environmental concerns into traditional accounting frameworks such as management accounting and EMA (Schaltegger et al., 2008).  Vietnam's rapid industrialization and economic growth have exacerbated environmental challenges, compelling policymakers and businesses to prioritize sustainable practices. As the country strives to meet international sustainability commitments, the role of EMA becomes crucial for its ability to align financial strategies with environmental goals. However, despite its global recognition as a tool for improving both economic and environmental efficiency, the adoption of EMA in Vietnam remains limited. The lack of comprehensive frameworks, awareness, and regulatory incentives has hindered its implementation, particularly in the banking sector (BURRITT et al., 2021; Nguyen, 2022). Many nations prioritize environmental preservation and fostering green growth as key objectives in their pursuit of sustainability. To ensure a balance between economic development and environmental protection, numerous studies worldwide have affirmed that EMA is a valuable tool for gaining a better understanding and quantifying environmental issues in decision-making processes (Burritt et al., 2002). EMA provides essential information to businesses to minimize environmental impacts, improve both economic and environmental efficiency, and achieve sustainability (IFAC, 2005).  While global research has advanced EMA application, significant gaps exist in its integration within Vietnam's commercial banking sector. Unlike industrial enterprises, banks play a critical role in directing financial resources towards environmentally sustainable projects, making their adoption of EMA pivotal. However, existing studies have largely overlooked the sector-specific dynamics of Vietnamese banks, such as their potential to influence environmental outcomes through credit allocation and operational changes. For example, banks in Vietnam are in a unique position to fund environmentally sustainable projects, yet the lack of comprehensive EMA frameworks results in missed opportunities for aligning financial performance with environmental goals (Nguyen & Nguyen, 2023; Tseng, 2010). Additionally, there is limited research exploring how mediators like environmental costs, knowledge management, and green innovation amplify EMA's impact on performance, creating a critical knowledge gap that this study aims to address.  Furthermore, the Vietnamese banking sector is increasingly pressured to align with international sustainability standards. Global benchmarks, such as those set by the United Nations Environment Programme Finance Initiative (UNEP FI), require banks to adopt sustainable practices, yet Vietnamese banks lag behind in implementing EMA to meet these expectations. This delay not only affects their global competitiveness but also limits their ability to achieve long-term financial stability while addressing environmental challenges (Deb et al., 2023; Nguyen, 2022).  In addition, Vietnamese banks face distinct operational challenges that complicate EMA implementation. These include limited managerial expertise in environmental accounting, lack of technological support for tracking environmental costs, and inadequate stakeholder pressure compared to their counterparts in developed economies. Addressing these operational hurdles is critical to ensuring that banks not only meet international benchmarks but also leverage EMA to drive innovation and competitive advantage in a rapidly globalizing market (BURRITT et al., 2021; Tseng, 2010). Moreover, this study is particularly novel as it integrates sector-specific mediators—environmental costs, knowledge management, and green innovation—into the analysis of EMA’s impact on bank performance. These mediators have been underexplored in the Vietnamese context, despite their proven importance in global banking practices. By investigating their role, the study provides actionable insights for policymakers and banking professionals seeking to enhance both financial and environmental performance (Deb et al., 2023; Nguyen & Nguyen, 2023).  This study bridges these gaps by focusing on the Vietnamese banking sector, offering empirical insights into how EMA affects bank performance through mediators such as environmental costs, knowledge management, and green innovation. Unlike prior studies that have predominantly explored manufacturing contexts, this research highlights the strategic potential of EMA in enabling banks to meet sustainability goals while enhancing bank performance. The research is particularly relevant as Vietnamese banks face increasing pressure to comply with global environmental standards and adopt greener operational models (BURRITT et al., 2021; Nguyen, 2022).  Therefore, this study aims to estimate the effect of EMA on bank performance in the context of the mediating role of environmental costs. To achieve this objective, the following questions need to be addressed: What are the specific mechanisms through which EMA influences bank performance in Vietnam, and how do environmental costs, knowledge management, and green innovation act as mediators? |
| 2. | Literature Review: | The author has revised the Literature review to make it relevant to hypothesis development and reduced redundant definitions: - **Page 4 – 6**. |
|  | o The literature review is extensive but occasionally redundant, especially in repeating the definitions of EMA and EC.  o Recommendation: Streamline the review to focus on critical theories and studies directly relevant to the hypotheses. | 2.1. Literature review  2.1.1. Legitimacy theory  Legitimacy theory provides a framework for understanding how organizations justify their operations in alignment with societal expectations. It emphasizes the necessity for businesses to disclose information on social, economic, and environmental aspects to maintain their legitimacy (Schiopoiu Burlea & Popa, 2013). EMA practices emerge as a strategic tool under this theory, helping organizations enhance their reputation, fulfill regulatory requirements, and secure societal approval (Gray et al., 1996). By disclosing environmental information through EMA, firms demonstrate accountability and commitment to social norms, addressing critical expectations of regulators, investors, and customers. These efforts not only safeguard legitimacy but also position organizations as socially responsible entities. This is particularly critical for Vietnamese banks, which face increasing scrutiny from both regulators and international stakeholders (T. H. Nguyen, 2022).  2.1.2. Contingency theory  Contingency theory underscores the need for organizations to adapt their management systems to fit their unique operational contexts. The appropriateness of EMA depends on factors such as industry type, organizational size, and strategic priorities (Bennett et al., 2002; Chenhall, 2009). For Vietnamese banks, the integration of EMA practices must address sector-specific challenges like limited technological infrastructure, evolving regulatory demands, and the drive for sustainable development (Deb et al., 2023; Nguyen T. H. & Nguyen, 2023). Contingency theory justifies tailoring EMA frameworks to include environmental costs and green innovation, ensuring that management accounting practices align with organizational goals while addressing external pressures for sustainability. This perspective reinforces the importance of flexibility in EMA adoption to enhance both environmental and financial outcomes.  2.1.3. Stakeholder theory  Stakeholder theory emphasizes the impact of diverse stakeholder groups on organizational decision-making processes and adherence to environmental laws (Deegan & Gordon, 1996; Freeman, 1984). This theory highlights the critical role of stakeholder engagement in promoting transparency and accountability through environmental cost management. Firms adopting EMA demonstrate their commitment to balancing stakeholder interests with environmental responsibilities, which can enhance trust, corporate reputation, and long-term viability. By integrating environmental accountability into their operations, organizations address stakeholder demands for responsible business practices and improved environmental outcomes. Vietnamese banks, in particular, are under increasing pressure from global initiatives like the United Nations Principles for Responsible Banking to align their operations with sustainability goals (UNEP FI., 2023). By integrating EMA into their practices, these banks can better meet stakeholder expectations while advancing their strategic objectives. |
| 3. | Research Methodology: |  |
|  | o The sample size of 312 is adequate but might benefit from more diversity in bank types or geographical representation. | Thank you for your insightful feedback regarding the sample size and its potential limitations. While the sample size of 312 respondents meets the methodological requirements for Partial Least Squares Structural Equation Modeling (PLS-SEM) as per Hair et al. (2017), we acknowledge the importance of sample diversity in enhancing the robustness and generalizability of research findings.  Our sample primarily consisted of respondents from commercial banks in urban areas, with a focus on Ho Chi Minh City. This focus was driven by the concentration of banking activities and access to experts with knowledge of environmental management accounting (EMA). However, we recognize that this may not fully represent the experiences and practices of banks in rural regions or those with specialized business models, such as state-owned banks or agricultural financing institutions.  To address this limitation, we have explicitly discussed the potential impact of the sample's composition on the generalizability of our findings in the revised manuscript. Specifically, we note that banks in less developed areas or those with distinct operational focuses may exhibit different EMA practices or face unique challenges that were not captured in our study. We have also recommended future research to include a more geographically and institutionally diverse sample to validate and expand upon our findings. |
|  | o Recommendation: Discuss the potential impact of sample limitations on generalizability. | We have revised the Research Methodology section to include these considerations and have elaborated on the implications for generalizability in the Conclusions and Limitations section of the manuscript. We believe these additions address your concerns and provide a more balanced discussion of the study's scope and applicability.  **Page 20.** |
| 4. | Data Analysis and Presentation: |  |
|  | o While the statistical analysis is thorough, the presentation of results (e.g., VIF values, discriminator validity) can be made more accessible with clearer visual aids. | The author has included the graphical representations, such as path diagrams for VIF values and the PLS-SEM model – Page 17 and Page 18.    **Figure 3. VIF values for all constructs**  This bar chart from Figure 3 presents the VIF values for each construct. The dashed red line at 3.0 indicates the typical threshold for multicollinearity concerns, showing that all VIF values are within acceptable limits.  **Page 17.**    **Figure 4. Path diagram for PLS-SEM relationships**  **Page 18.**  As exhibited in Figure 4, EMA had a strong impact on KM (0.763), GI (0.750), and EC (0.579), indicating its critical role in the entire model. KM and GI also directly affect EC, but their coefficients are lower at 0.120 and 0.129, respectively, but they are significant contributing factors. Specifically, EC serves as both a dependent variable and a robust mediating variable, directly affecting BP with a path coefficient of 0.741, underscoring the fundamental construct of transforming environmentally friendly behavior into bank performance sustainability. The relationships indicate a clear directional hierarchy in which EMA drives KM, drives GI, enhances EC, and improves BP. Along with the summary statistics presented in Table 5, this figure provides an understanding of how the constructs interact to create a sustainable and competitive advantage. |
|  | o Recommendation: Include more graphical representations, such as path diagrams for the PLS-SEM model. |  |
| 5. | Conclusion and Practical Implications: |  |
|  | o The conclusion reiterates the findings but lacks a forward-looking perspective on future research and practical applications. | The author has revised and provided specific strategies for banks to implement EMA practices and suggest areas for further research based on the reviewer’s comments: - **Page 20 – 21**.  Based on the findings, we propose some suggestions for applying EMA in Vietnamese commercial banks. First, banks ought to integrate EMA frameworks in their strategic governance systems by creating in-house systems that capture and integrate environment cost data. Such tools consist of the use of advanced technologies (for example: data analytics, artificial intelligence) to help improve the reliability of environmental cost tracking and reporting. Second, the managerial know-how on environmental accounting should be augmented through training programs so that human resources, as a labour force becomes proficient in this innovative management technique. Third, the banks can fund green innovation projects, including energy-efficient technologies and sustainable product development, to emphasize their support of environmental sustainability.  Some findings are specific to this study; it should be aware of some limitations. First, the small sample size contributes to another limitation: the need for future research that can use a larger, more heterogeneous sample. Second, more research should be conducted exploring the long-term influence of EMA on financial stability and its broader consequences for society in developing countries like Vietnam. The research may also explore how digital transformation enables EMA adoption and the impact on new sustainability practices. Furthermore, investigating the role of regulatory and technological factors in adopting EMA among several banking systems would yield significant insight for adopting suitable practices. Finally, more mediators, such as corporate social responsibility and environmental risk management, may clarify the relationship between EMA and bank performance. |
|  | o Recommendation: Provide specific strategies for banks to implement EMA practices and suggest areas for further research. |  |
| 6. | 6Language and Formatting: |  |
|  | o There are minor grammatical errors and inconsistencies in formatting. | The author has revised and conducted language review. |
|  | o Recommendation: Conduct a thorough language review to ensure clarity and adherence to journal guidelines. |  |

| **No.** | **Reviewer 2’s comments** | **Author’s explanations** |
| --- | --- | --- |
| 1. | Introduction • The introduction lacks an adequate background that contextualizes the study. Key concepts related to environmental accounting and its relevance to the environmental impacts being examined are not discussed, leaving readers without a clear understanding of the study's foundation.  • A well-defined research problem is essential to frame the study, yet the manuscript does not articulate any specific problem. Without a clear problem statement, the motivation and necessity of the research are not evident.  • The manuscript does not include a meaningful review of prior studies. There is no critical analysis of existing research, no identification of gaps, and no explanation of how this study differs from or builds upon previous work. This oversight diminishes the manuscript’s scholarly contribution.  • The introduction does not articulate the theoretical or practical contributions of the research. It is unclear how this study adds value to the academic field or to practical applications in environmental accounting. • The research is conducted in the context of Vietnamese banks, yet there is no rationale for this choice. The study does not explain how banks contribute to or mitigate environmental impacts, which creates a significant disconnect between the topic and the case selection.  • The introduction relies on a limited number of references, many of which are outdated. The use of older references weakens the validity of the claims made and does not reflect the current state of research in the field. To substantiate the research arguments, the manuscript needs to draw from a broader and more recent body of literature. | The author has revised the Introduction section based on the reviewer’s suggestions:  + Added context on EMA’s role: Expanded discussion on EMA’s importance in integrating environmental concerns into financial decision-making.  + Explained why the Vietnamese banking sector is a significant context for examining EMA.  + Gap identification: Emphasized the limited research on EMA in the banking sector.  + Linked the study to international sustainability benchmarks like UNEP FI.  + Then, the author has clearly defined the research objective and questions to frame the study.  **1. Introduction – Page 2**  Environmental pollution resulting from industrial activities is becoming increasingly severe. Methods for mitigating environmental pollution in business operations are of great concern to both enterprises and regulatory authorities. To address this issue, businesses need to understand the regulations, invest in upgrading waste treatment systems, and simultaneously implement environmental management accounting (EMA). Sustainable development is a process that meets current needs without compromising the interests of future generations. As a crucial and leading component of sustainable economic development, businesses are responsible for assessing and accounting for environmental factors to protect the environment.  The environment has significant importance in the realm of sustainable development. Organizations are increasingly prioritizing sustainable accounting, aiming to integrate environmental concerns into traditional accounting frameworks such as management accounting and EMA (Schaltegger et al., 2008).  Vietnam's rapid industrialization and economic growth have exacerbated environmental challenges, compelling policymakers and businesses to prioritize sustainable practices. As the country strives to meet international sustainability commitments, the role of EMA becomes crucial for its ability to align financial strategies with environmental goals. However, despite its global recognition as a tool for improving both economic and environmental efficiency, the adoption of EMA in Vietnam remains limited. The lack of comprehensive frameworks, awareness, and regulatory incentives has hindered its implementation, particularly in the banking sector (BURRITT et al., 2021; T. H. Nguyen, 2022). Many nations prioritize environmental preservation and fostering green growth as key objectives in their pursuit of sustainability. To ensure a balance between economic development and environmental protection, numerous studies worldwide have affirmed that EMA is a valuable tool for gaining a better understanding and quantifying environmental issues in decision-making processes (Burritt et al., 2002). EMA provides essential information to businesses to minimize environmental impacts, improve both economic and environmental efficiency, and achieve sustainability (IFAC, 2005).  While global research has advanced EMA application, significant gaps exist in its integration within Vietnam's commercial banking sector. Unlike industrial enterprises, banks play a critical role in directing financial resources towards environmentally sustainable projects, making their adoption of EMA pivotal. However, existing studies have largely overlooked the sector-specific dynamics of Vietnamese banks, such as their potential to influence environmental outcomes through credit allocation and operational changes. For example, banks in Vietnam are in a unique position to fund environmentally sustainable projects, yet the lack of comprehensive EMA frameworks results in missed opportunities for aligning financial performance with environmental goals (Nguyen T. H. & Nguyen, 2023; Tseng, 2010). Additionally, there is limited research exploring how mediators like environmental costs, knowledge management, and green innovation amplify EMA's impact on performance, creating a critical knowledge gap that this study aims to address.  Furthermore, the Vietnamese banking sector is increasingly pressured to align with international sustainability standards. Global benchmarks, such as those set by the United Nations Environment Programme Finance Initiative (UNEP FI), require banks to adopt sustainable practices, yet Vietnamese banks lag behind in implementing EMA to meet these expectations. This delay not only affects their global competitiveness but also limits their ability to achieve long-term financial stability while addressing environmental challenges (Deb et al., 2023; T. H. Nguyen, 2022).  In addition, Vietnamese banks face distinct operational challenges that complicate EMA implementation. These include limited managerial expertise in environmental accounting, lack of technological support for tracking environmental costs, and inadequate stakeholder pressure compared to their counterparts in developed economies. Addressing these operational hurdles is critical to ensuring that banks not only meet international benchmarks but also leverage EMA to drive innovation and competitive advantage in a rapidly globalizing market (BURRITT et al., 2021; Tseng, 2010). Moreover, this study is particularly novel as it integrates sector-specific mediators—environmental costs, knowledge management, and green innovation—into the analysis of EMA’s impact on bank performance. These mediators have been underexplored in the Vietnamese context, despite their proven importance in global banking practices. By investigating their role, the study provides actionable insights for policymakers and banking professionals seeking to enhance both financial and environmental performance (Deb et al., 2023; Nguyen T. H. & Nguyen, 2023).  This study bridges these gaps by focusing on the Vietnamese banking sector, offering empirical insights into how EMA affects bank performance through mediators such as environmental costs, knowledge management, and green innovation. Unlike prior studies that have predominantly explored manufacturing contexts, this research highlights the strategic potential of EMA in enabling banks to meet sustainability goals while enhancing bank performance. The research is particularly relevant as Vietnamese banks face increasing pressure to comply with global environmental standards and adopt greener operational models (BURRITT et al., 2021; T. H. Nguyen, 2022).  Therefore, this study aims to estimate the effect of EMA on bank performance in the context of the mediating role of environmental costs. To achieve this objective, the following questions need to be addressed: What are the specific mechanisms through which EMA influences bank performance in Vietnam, and how do environmental costs, knowledge management, and green innovation act as mediators?  **Page 4.** |
| 2. | Literature Review:  The section on prior studies is overly detailed, the extensive focus on the literature review appears to have come at the expense of other essential sections, such as methodology and discussion. These sections lack sufficient detail and depth, which undermines the overall quality of the manuscript. | The author has revised the Literature review section and linked it to the hypotheses – **Page 4 – 5.**  **2.1. Literature review**  **2.1.1. Legitimacy theory**  Legitimacy theory provides a framework for understanding how organizations justify their operations in alignment with societal expectations. It emphasizes the necessity for businesses to disclose information on social, economic, and environmental aspects to maintain their legitimacy (Schiopoiu Burlea & Popa, 2013). EMA practices emerge as a strategic tool under this theory, helping organizations enhance their reputation, fulfill regulatory requirements, and secure societal approval (Gray et al., 1996). By disclosing environmental information through EMA, firms demonstrate accountability and commitment to social norms, addressing critical expectations of regulators, investors, and customers. These efforts not only safeguard legitimacy but also position organizations as socially responsible entities. This is particularly critical for Vietnamese banks, which face increasing scrutiny from both regulators and international stakeholders (T. H. Nguyen, 2022).  **2.1.2. Contingency theory**  Contingency theory underscores the need for organizations to adapt their management systems to fit their unique operational contexts. The appropriateness of EMA depends on factors such as industry type, organizational size, and strategic priorities (Bennett et al., 2002; Chenhall, 2009). For Vietnamese banks, the integration of EMA practices must address sector-specific challenges like limited technological infrastructure, evolving regulatory demands, and the drive for sustainable development (Deb et al., 2023; Nguyen T. H. & Nguyen, 2023). Contingency theory justifies tailoring EMA frameworks to include environmental costs and green innovation, ensuring that management accounting practices align with organizational goals while addressing external pressures for sustainability. This perspective reinforces the importance of flexibility in EMA adoption to enhance both environmental and financial outcomes.  **2.1.3. Stakeholder theory**  Stakeholder theory emphasizes the impact of diverse stakeholder groups on organizational decision-making processes and adherence to environmental laws (Deegan & Gordon, 1996; Freeman, 1984). This theory highlights the critical role of stakeholder engagement in promoting transparency and accountability through environmental cost management. Firms adopting EMA demonstrate their commitment to balancing stakeholder interests with environmental responsibilities, which can enhance trust, corporate reputation, and long-term viability. By integrating environmental accountability into their operations, organizations address stakeholder demands for responsible business practices and improved environmental outcomes. Vietnamese banks, in particular, are under increasing pressure from global initiatives like the United Nations Principles for Responsible Banking to align their operations with sustainability goals (UNEP FI., 2023). By integrating EMA into their practices, these banks can better meet stakeholder expectations while advancing their strategic objectives. |
| 3. | Methodology Lack of Clarity in the Mixed-Method Approach: • While the authors claim to have adopted a mixed-methods approach that integrates qualitative and quantitative research techniques, the steps for conducting the qualitative component are not clearly outlined. The absence of detailed procedures, such as data collection, sampling, or coding methods, creates ambiguity and casts doubt on the reliability of the qualitative findings. • According to Hair et al., (2017), Structural Equation Modeling (SEM) consists of two main components: • Measurement Model: This part describes the relationship between latent variables (constructs that are not directly observable) and their corresponding observed indicators. It allows researchers to assess the validity and reliability of the constructs. • Structural Model: This part illustrates the causal relationships between the latent variables, showing how they influence one another based on theoretical assumptions. • The authors have ignored the important part of the measurement of the model which is Factor loadings. Factor loadings of ≥ 0.70 contribute to convergent validity, demonstrating that the indicators of a construct are highly related to one another. This ensures that the construct is well-defined by its indicators. The ignoring of this part leads to invalid results in the structural model. • In addition, the structural model lacks a lot of information that must be presented to ensure the validity of the results, such as standard deviation, Confidence Interval, and t-value.  • In general, the methodology is unclear and full of ambiguity, which requires a complete reconsideration. | We acknowledge that the initial description of our mixed-methods approach lacked clarity regarding the qualitative component. To address this, we have revised the **Methodology** section to provide a detailed explanation of the procedures used in the qualitative phase – **Page 12 – 13**.   1. **Data Collection**: We conducted semi-structured interviews with 10 senior banking professionals, including finance managers, chief accountants, and heads of departments, to explore their perspectives on environmental management accounting (EMA). 2. **Sampling**: Participants were selected using purposive sampling to ensure representation from individuals with extensive knowledge and experience in EMA practices in commercial banks. 3. **Coding and Analysis**: The interview transcripts were thematically analyzed using an inductive approach, allowing patterns and themes to emerge organically.   Regarding the SEM analysis, we agree with the reviewer’s observation that important elements such as factor loadings, standard deviations, confidence intervals, and t-values were not explicitly presented in the manuscript. We have addressed these gaps in the following ways:   1. **Measurement Model**:    - We have included a detailed table presenting the factor loadings for each indicator. All loadings were checked against the threshold of ≥0.70 to confirm convergent validity, as suggested by Hair et al. (2017). These results demonstrate that the constructs are well-defined by their respective indicators.    - Composite reliability and average variance extracted (AVE) for all constructs were already reported in the manuscript and confirmed their validity. 2. **Structural Model**: - **Page 17**.    - We have updated the results section to include the missing statistics:      - **Standard deviations**: To show the variability in estimates.      - **Confidence intervals (95%)**: To provide a range of plausible values for the model parameters.      - **t-values**: To evaluate the statistical significance of relationships.    - These additional statistics provide greater transparency and support the reliability of the structural model results. |
| 4. | Issues in the Discussion Section • The discussion is written descriptively without a clear link between the results and the research questions. The findings are presented, but there is little effort to explain how they address the core questions of the study or how they contribute to understanding the research problem. This disconnect weakens the paper's coherence and its ability to advance knowledge in the field. • The discussion section does not adequately compare or contrast the findings with existing research. There is no reference to how the results align with or differ from previous studies, which is essential for contextualizing the contribution of the current research. A thorough comparison with past studies would highlight the novel aspects of this research and its relevance to the field. • The discussion does not explicitly address the theoretical contributions of the research. The study’s implications for theory are not discussed, nor is there an explanation of how the findings contribute to the development or refinement of the theoretical framework used in the study. Without this discussion, the theoretical significance of the research remains unclear. | The author has revised the Discussion section based on the reviewer’s comments:  + Making the link to research questions: by connecting the findings to address the core research questions and hypotheses.  + Comparing with prior studies: Highlighted alignments and novel contributions, emphasizing the uniqueness of the banking context.  + Theoretical contributions in contingency theory and stakeholder theory.  + Emphasis on novelty: Highlighted the originality of exploring EMA in the Vietnamese banking sector and incorporating mediators.    **Page 18 – 20**.  As exhibited in Figure 4, EMA had a strong impact on KM (0.763), GI (0.750), and EC (0.579), indicating its critical role in the entire model. KM and GI also directly affect EC, but their coefficients are lower at 0.120 and 0.129, respectively, but they are significant contributing factors. Specifically, EC serves as both a dependent variable and a robust mediating variable, directly affecting BP with a path coefficient of 0.741, underscoring the fundamental construct of transforming environmentally friendly behavior into bank performance sustainability. The relationships indicate a clear directional hierarchy in which EMA drives KM, drives GI, enhances EC, and improves BP. Along with the summary statistics presented in Table 5, this figure provides an understanding of how the constructs interact to create a sustainable and competitive advantage.  Similarly, from Table 5, this study examines the role of EMA in improving bank performance through mediating factors such as knowledge management (KM), green innovation (GI), and environmental costs (EC). The findings reveal that EMA significantly enhances KM, GI, and EC, which, in turn, positively affect bank performance. These results provide robust evidence supporting the research hypotheses and addressing the core research questions by demonstrating the mechanisms through which EMA exerts its influence.  The study directly answers the research questions by elucidating the pathways linking EMA to bank performance. The results indicate a strong positive relationship between EMA and KM (β = 0.763, p < 0.001) as well as GI (β = 0.750, p < 0.001), underscoring EMA’s ability to foster knowledge-sharing and innovative practices within banks. These findings highlight that EMA is not merely a financial tool but also a strategic enabler that drives organizational innovation and knowledge management capabilities. Furthermore, the strong impact of EC on bank performance (β = 0.741, p < 0.001) emphasizes the importance of environmental cost management as a key mediator, confirming its critical role in bridging sustainability initiatives with financial outcomes. By revealing these mechanisms, the study offers empirical validation for the conceptual model and enhances our understanding of how sustainability practices create value in the banking sector.  These findings are consistent with the literature while also providing new insights. EMA's observed effect on KM aligns with previous research by (Deb et al., 2023; Kapiyangoda & Gooneratne, 2021; Mansoor et al., 2021) within manufacturing contexts. However, this study explores the banking industry, where structural differences in financial operations exist. Likewise, the connection between EMA and GI confirms the findings reported by (Hadj, 2020; Saeidi et al., 2018), who highlighted that EMA complements green innovation. In contrast, by narrowing the scope to the Vietnamese banking sector, this research emphasizes the untapped potential of the banking sector as an environmentally pertinent source of credit allocation and resource mobilization, which are parameters frequently disregarded in previous literature. This small contribution highlights the importance of EMA as a mechanism to reconcile financial outcomes with sustainability targets, with a focus on emerging economies. Following that, this study examines whether the EC enhances the performance of commercial banks in Vietnam. The direct effect of EC on the performance of companies has been explored by (Lee & Suh, 2022) while the research examines whether EMA affects bank performance under the mediating role of EC.  This study makes important theoretical contributions by refining contingency theory and stakeholder theory, specifically within the context of EMA. The effectiveness of EMA is contingent upon industry-specific factors such as the technological and regulatory environment of Vietnamese banks, lending support to contingency theory. The results show the need to integrate EMA schemas into the local context to harness the most benefit. Stakeholder theory is again supported by demonstrating how transparency and environmental accountability–underlined by strong EMA–lead stakeholders to have more stake in the company, which, in turn, feeds into the financial performance of the company. Advancing theoretical perspectives on the realization of EMA in various organizations and ecological modalities in this manner leads to a broad-based platform for the applications of EMA. |
| 5. | • The practical implications of the research are not addressed in the discussion. Given the applied nature of the study, it is crucial to outline how the findings can inform practice in the field, such as in environmental accounting or bank management. The lack of practical insights limits the relevance of the study to practitioners and policymakers. These issues significantly weaken the manuscript’s contribution to both theory and practice. The discussion needs to be restructured to clearly link the findings to the research questions, engage with the relevant literature, and outline both theoretical and practical contributions. | The author has added practical implications based on the reviewer’s comments:  **Page 20**.  Based on the findings, we propose some suggestions for applying EMA in Vietnamese commercial banks. First, banks ought to integrate EMA frameworks in their strategic governance systems by creating in-house systems that capture and integrate environment cost data. Such tools consist of the use of advanced technologies (for example: data analytics, artificial intelligence) to help improve the reliability of environmental cost tracking and reporting. Second, the managerial know-how on environmental accounting should be augmented through training programs so that human resources, as a labour force becomes proficient in this innovative management technique. Third, the banks can fund green innovation projects, including energy-efficient technologies and sustainable product development, to emphasize their support of environmental sustainability. |
